# The Responsibilities of Voluntary Hospitals

**Published:** 1916-12-30

**Authors:** 


### THE RESPONSIBILITIES OF VOLUNTARY HOSPITALS.

By A PROVINCIAL SUPERINTENDENT.

Attention is particularly drawn to the distribution proceedings of King Edward's Fund published in The Hospital last week. The report is remarkable at once for the fact that the total grants distributed are the largest in the history of the Fund. But the point we particularly emphasise is the allusion of Lord Iveagh to the future needs and responsibilities of the hospitals. "There is no doubt," he said, "that the needs of the hospitals will continue to grow in the near future. The burden of 1916 will inevitably be greater; and, if the Fund is to continue to do its part, an increased distribution may become necessary." He commended the consideration of these needs to those who forget that the demands on the hospitals were proportionately greater, and in the present crisis continually growing.

### An Increasing Weight of Responsibility.

The Hospital has repeatedly drawn attention to these growing needs and to the generous way in which the voluntary institutions have responded to the insistent demands upon them since the outbreak of war. There is not a hospital of any size which has not willingly rendered national service by offering beds for wounded soldiers to supplement the accommodation of military hospitals throughout the country. In the supply of physicians and surgeons, resident medical officers, and nurses, hospitals have played a very important part: they have, in fact, been depleted of their highly trained and experienced officers, and are carrying on their work with what in normal times would be considered an undue proportion of less experienced but most willing workers. This is especially the case with nursing staffs, the proportion of probationers to fully trained workers being very much greater than at any previous period. In this respect the Government itself are quickening the pace, and incidentally increasing the disproportion by the increased scale of pay offered to members of Queen Alexandra's Imperial Military Nursing Service and the Territorial Nursing Service, as well as to V.A.D. workers and special probationers in military hospitals, and by augmenting the allowances for washing, etc. We pointed out in a recent issue that there was nothing unfair in these regulations, nor was the War Office singular in having to grant increased rates of pay to nurses, which were everywhere on the up grade. We mentioned that at the London Hospital the increased rates of pay recently adopted would entail an additional expenditure of £4,000 per annum, an expenditure which would be proportionately great in all hospitals according to the size of their staffs. This is the financial side of the problem. There is, however, an indirect influence which will not unlikely bear heavily upon institutions. The increased scale of Government pay may attract a number of the already lessened staff of fully-trained nurses in hospitals unless a similar scale is adopted by institutions, and the more attractive rates of pay to V.A.D. workers and temporary probationers in military hospitals may lessen the number of candidates for general training in hospitals. The difficulties of administration must therefore undoubtedly increase.

### Pay Your Way, War Office!

The columns of The Hospital have recently given ample instances of the immense increases in the prices of food, drugs, dressings, and all purchasable commodities, and of the difficulties of obtaining adequate supplies. The outlook, indeed, is for seriously increased responsibilities all round, and we therefore emphasise Lord Iveagh's plea for a fuller consideration of the increasing claims. This fuller consideration is also commended to the War Office. The rate of pay approved of at the beginning of the war for the reception of wounded soldiers (3s. to 4s. per day, according to circumstances and to local conditions) bears no real relationship to the present actual cost of maintenance, and the payment of less than the actual cost means an addition to the financial strain which institutions should not be asked to bear. A 50 per cent. increase on the present War Office scale of remuneration for soldier in-patients is certainly warranted. We again particularly emphasise the increasing needs of the hospitals, and their special claims this year to a larger share of the charity of the public at this season of goodwill.

